# Leveraging physical intelligence for the self-design of high performance engineering structures

**DOI:** 10.1038/s41598-022-15229-z

**Published:** 2022-07-08

**Authors:** Jessé Paixão, Emeline Sadoulet-Reboul, Emmanuel Foltête, Gaël Chevallier, Scott Cogan

**Affiliations:** University Bourgogne Franche-Comté, FEMTO-ST Institute, CNRS/UFC/ENSMM/UTBM, Department of Applied Mechanics, 24 chemin de l’Epitaphe, 25000 Besançon, France

**Keywords:** Engineering, Aerospace engineering, Mechanical engineering

## Abstract

The design of complex engineering structures largely relies on *computational intelligence* in the form of science-based predictive models to support design decisions. This approach requires modeling and manufacturing uncertainties to be accounted for explicitly and leads to an inescapable trade-off of performance for robustness. To remedy this situation, a novel *self-design* paradigm is proposed that closes the loop between the design and manufacturing processes by leveraging *physical intelligence* in the form of real-time experimental observations. This allows the real-time product behavior to participate in its own design. The main benefit of the proposed paradigm is that both manufacturing variability and difficult-to-model physics are accounted for implicitly via in situ measurements thus circumventing the performance-robustness trade-off and guaranteeing enhanced performance with respect to standardized designs. This paradigm shift leads to tailored design realizations which could benefit a wide range of high performance engineering applications. The proposed paradigm is applied to the design of a simply-supported plate with a beam-like absorber introduced to reduce vibrations based on an equal peaks performance criteria. The experimental setup includes a low-cost 3D printer driven by a simple decision algorithm and equipped with an online vibration testing system. The performances of a small population of self-designed plates are compared to their standardized counterparts in order to highlight the advantages and limitations of the new self-design manufacturing paradigm.

## Introduction

The current state-of-practice in engineering design leverages computational intelligence in the form of science-based predictive modeling to provide decision support for the early to final design phases. Uncertainty due to manufacturing tolerances, environmental conditions, and most importantly in the physics-based models themselves, has led to the development of model-based robust design methods to ensure statistically acceptable performances of the manufactured products^1^. The main steps of this design paradigm can be summarized as follows: (1) define the design space with its design variables, objectives, constraints, and uncertainty models; (2) leverage high fidelity physics-based models to specify product design providing acceptable performance under uncertainty; and (3) manufacture nominally identical or standardized products to design specifications. While this paradigm is omnipresent in engineering applications where complex and generally multiphysical systems must be designed and fabricated, it has a number of important drawbacks: the necessity of accurate modelling of all dominant sources of uncertainty to ensure reliable performance^2^; the high cost of reducing manufacturing tolerances and modeling errors; an unavoidable trade-off between performance and robustness to uncertainty^3^. The last point is of particular importance for high performance applications since for a given candidate design, changes introduced to guarantee acceptable performance under uncertainty translate to suboptimal designs with degraded performance. Moreover, it is very difficult to satisfy equality design constraints in a robust-design approach.

A novel self-design paradigm is proposed herein allowing the above limitations to be circumvented by closing the loop between the design and manufacturing process by leveraging physical intelligence to guide in situ design changes. The introduction of feedback loops in engineering applications is of course not new. Simple mechanical devices (e.g. water level control or centrifugal speed control^4^), active controllers (e.g. shape control^5^), metamaterials^6^ , and self-programming networks^7^, are just a few examples that implement closed loop processes to attain a sought after functionality. Living organisms display a rich variety of self-regulating or self-adapting processes, including homostasis^8^, thigmomorphogenesis in trees growth^9^, and Wolff’s law for bone growth^10^ to name but a few. These biological mechanisms are the embodiment of physical intelligence wherein information concerning the local state of an organism drives biological processes (e.g. targeted cell growth) to better meet the organisms performance requirements (e.g. zero stress gradients). Although physical intelligence has been observed in many living organisms, this concept has only recently been explored for engineering application, particularly focused in the robotic field for creation of autonomous machines. Sitti^11^ defined it as ‘physically encoding sensing, actuation, control, memory, logic, computation, adaptation, learning and decision-making into the body of an agen’. In the proposed paradigm, physical intelligence is derived from in-situ measurements and leveraged to drive with hardware-in-the-loop the manufacturing process.

**Figure 1:**
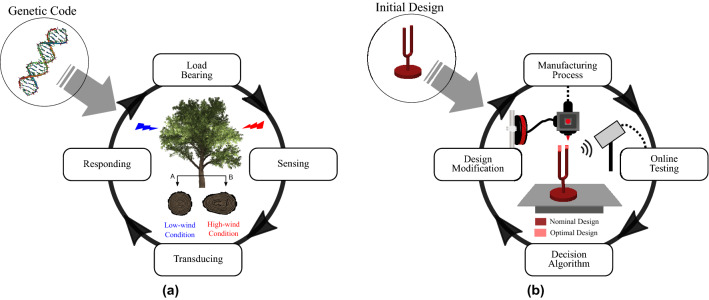
Schematic representation of the self-design manufacturing paradigm inspired by the biological mechanism of mechanosensitive control of plant growth. (**a**) Moulia^14^ presented a theoretical division of plants response to mechanical loads into four processes: load bearing, sensing, transducing and responding. (**b**) Similarly, the self-design manufacturing paradigm can be divided into four processes: manufacturing, online testing, decision algorithm, and design modification.

Plants in particular have a special potential to respond to changing environmental conditions during the development and formation of roots, stems, branches, leaves, and flowers. Despite being genetically encoded, they have a developmental plasticity that is critical for their survival as sessile organisms^12^. For example, genetically identical trees growing under different conditions of wind exposure will present stem thickening on the leeward side^13^. Moulia et al.^14^ investigated the mechanisms underlying this mechanosensitive control of plant growth and broke it into the four processes illustrated in Fig. 1a. The analogy for engineering applications is attained by closing the loop between the design and manufacturing processes by allowing real-time product behavior to participate in its own design, and consequently by providing a developmental plasticity. The proposed bio-inspired self-design paradigm focuses here on the load bearing structural components of a complex engineering system and it can be similarly divided into the four processes presented in Fig. 1b, summarized as follows: (1) manufacturing process—fabricate the nominal design; (2) online testing—combine the manufacturing process with in situ testing—the physical intelligence—to drive real physical design modifications; (3) decision algorithm—evaluate the system performance and decide to continue or stop the loop; (4) design modification—propose the modification of the design based on data from the online testing to improve the system’s performance.

The proposed self-design paradigm starts with an initial nominal design, which is modified along the closed-loop process, in order to manufacture tailored products with enhanced performance with respect to their standardized counterparts. In contrast to the model-based design approach, the main advantages of the self-design paradigm are: combine mass production approach with physical intelligence via real-time in situ performance measurements to yield optimal performances; circumvents robustness-performance trade-off by taking into account the true real-time behavior of the structure that implicitly depends on the specific realization of a statistical population under test; alleviates the need for high fidelity models; guarantees enhanced performance with respect to a robust design based on computational intelligence; and leads to unique tailored designs. The bio-inspired self-design manufacturing paradigm proposed in this work is aligned with perspectives of the so-called fourth industry revolution which takes advantage of information from both the virtual and physical worlds (cyber-physical systems) combined with advanced manufacturing technologies to achieve flexible, smart, and reconfigurable manufacturing processes for the production of high-performance individualized products^15,16^. Therefore, we propose a new strategy for producing high-performance systems that circumvents the traditional practice of model-based design under uncertainty by directly observing the in-situ system response and adapting the design accordingly. This paradigm is particularly attractive in the current technological environment with availability of advanced information flow from cyber-physical systems and emerging manufacturing technologies. Additive manufacturing is particularly promising due to its versatility and ability to fabricate a product from scratch by continually adding material. However, uncertainties concerning the quality of additive manufactured products severely compromise its adoption in many industry sectors^17^. In this context, given the relatively high material and geometric uncertainties associated with this process, the proposed self-design paradigm is very attractive. Herein, we propose a demonstration case involving the design and additive manufacturing of a structure with an integrated device for vibration attenuation, a vibration absorber, whose performance is known to be very sensitive to mistuning. To the authors’ knowledge, constraints on structural dynamic performance have not yet been introduced within a closed-loop design-manufacturing process.

### Demonstration case

In order to demonstrate the potential of the self-design paradigm we explored the problem of vibration attenuation in mechanical structures. Although has been intensively explored over the last century it remains an important challenge in engineering applications ranging from machinery to satellites^18^. A traditional method for suppressing vibration is through the use of vibration absorbers. This type of device was proposed by Frahm^19^ to reduce the rolling motion of ships and essentially consists of an undamped mass-spring system, the absorber also called dynamic vibration absorber, whose parameters may be tuned at a specific frequency to attenuate the motion or vibration of the host structure where the device is connected. Later, Ormondroyd and Den Hertog^20^ proposed to add a damper element to improve the vibration attenuation of the absorbers. In this case, the vibration absorber is commonly named as tuned mass damper. Vibration absorbers are especially useful for mitigating narrow bandwidth vibration, and they must be designed in order to finely tune the operating frequency. Although, they have been used in numerous real-life structures due to their simplicity, effectiveness and low-cost, this kind of device presents a major drawback related to the high sensitivity to mistuning, which can degrade their efficiency in the presence of uncertainty and limit their application in the context of high-performance structures^21^.

The effectiveness of a vibration absorber relies on tuning its resonance frequency and damping properties for a given primary structure, such that significant kinetic energy is transferred from the vibrating primary structure to the absorber. A very insightful real-life case concerning absorber tuning is found in the application of tuned mass dampers for the attenuation of lateral wind-induced vibration in tall buildings, such as Chifley Tower, Taipei 101 and Shanghai Tower^22,23^. In this type of application, a pendulum-like vibration absorber consisting of a suspended mass is installed close to the top of the building. Although the device is designed based on a numerical model to achieve a maximum vibration attenuation of the fundamental lateral mode shape, due to the absorber high-sensitivity to mistuning and uncertainties related to the model and construction/installation of the device, a frequency adjustable mechanism is necessary. To make the absorber meet the expected design requirements in the installation process, the suspended mass is lifted with cables whose lengths are adjustable to ensure that the absorber frequency is consistent with that of the main structure. This adjustable mechanism allows a finely tuning of the absorber resonance frequency iteratively going from an initial cable length to a modified cable length to achieve an acceptable vibration attenuation performance.

**Figure 2:**
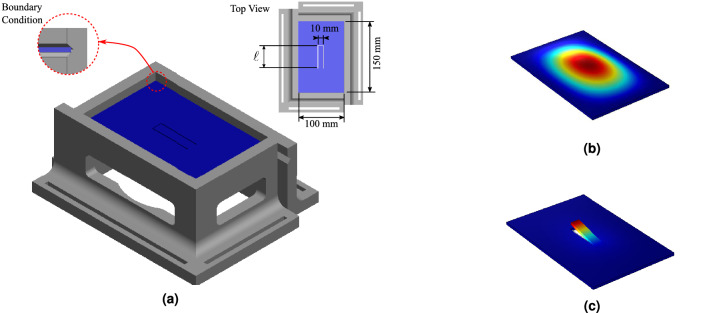
(**a**) 3D-view of the plate with beam-like absorber and the support structure and the first vibration mode shapes of the plate without (**b**) and with the beam-like absorber (**c**).

We propose here a demonstration case consisting in the design and manufacturing of a beam-like absorber inserted into a plate for the vibration attenuation of its first mode, shown in Fig. 2a. Similar structures have been explored recently in the literature^24–26^.The finite element simulation of the first mode of vibration of the simply-supported plate without and with the beam-like vibration absorber, shown in Fig. 2b, c, illustrates the effects of vibration attenuation caused by the insertion of this device into the host structure, which by destructive interference attenuates the targeted resonance frequency at the expense of two additional resonances, and a high amplitude of vibration of the absorber^27^.

### Vibration absorber tuning

**Figure 3:**
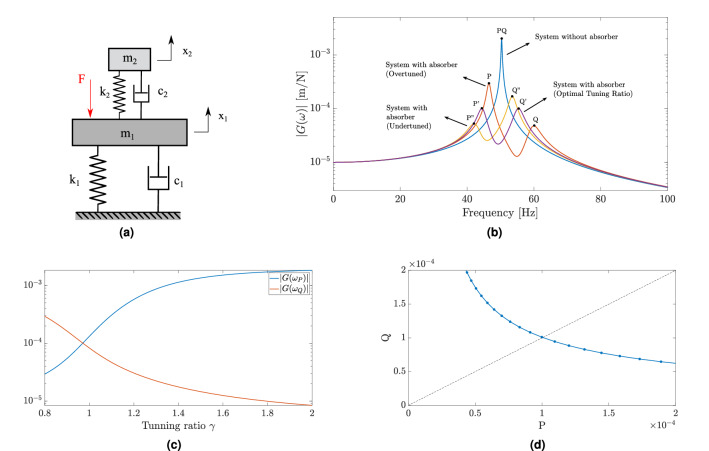
(**a**) One-dimensional lumped mass model of host structure coupled with a vibration absorber. (**b**) FRF for the primary system without the absorber (blue) and with the absorber considering different tuning ratios: undertuned (orange), optimal (purple) and overtuned (yellow). The two-peaks are identified sequentially as P and Q for the FRF of the system with the absorber and as PQ for the single peak for the FRF of the system without the absorber. (**c**) FRF peaks P and Q of the primary system versus tuning ratio $\gamma$. (**d**) Representation of FRF peaks into PQ space for tuning ratio varying from 0.8 to 1.2 .

This section briefly reviews the theory of vibration absorber tuning using a simple lumped mass model of the proposed demonstration case. The first vibration mode of the system, consisting in the plate with the beam-like absorber, can be modeled as two mass-spring-damper systems connected in series as shown in Fig. 3a. The larger mass represents the host structure or primary system, the plate, and the smaller mass represents the absorber. The equations of motion in the frequency domain of the coupled system are:

1 $$\begin{aligned} \left[ \begin{array}{ll} k_{1}+k_{2}+i \omega \left( c_{1}+c_{2}\right) -\omega ^{2} m_{1} &{} -\left( k_{2}+i \omega c_{2}\right) \\ -\left( k_{2}+i \omega c_{2}\right) &{} k_{2}+i \omega c_{2}-\omega ^{2} m_{2} \end{array}\right] \left\{ \begin{array}{l} X_{1} \\ X_{2} \end{array}\right\} =\left\{ \begin{array}{c} F(\omega ) \\ 0 \end{array}\right\} \end{aligned}$$

where $m_1$, $k_1$ and $c_1$ denote the mass, spring and damper of the host structure; $m_2$, $k_2$ and $c_2$ their analagous of the absorber; and $X_1(\omega )$ and $X_2(\omega )$ are the displacements of the harmonically-forced host system and of the absorber.

The design of the absorber for vibration attenuation of a specific mode can be formalized as an optimization problem involving the minimization of the maximum amplitude ratio of the primary system’s response to the excitation force, i.e., $G(\omega ) = X_1(\omega )/F(\omega )$, the receptance. Although there is still a small amount of material damping in the beam-like absorber, which is inherent to the whole structure and represented in the lumped mass model by the damper elements, it can not be controlled for the vibration absorber tuning. This system behaves then more like an undamped vibration absorber than a tuned mass damper. Thus, if we consider the dampers of the system fixed and the mass and stiffness of the absorber the variables to be tuned. the tuning ratio between the resonance frequencies of the absorber and the primary system can be used as the design variable and the optimization problem is posed as follows:

2 $$\begin{aligned} \gamma ^{\star } = \arg \left[ \min _{\gamma \in \mathbb {R}^{+}}\Vert G\left( \omega \mid m_1, c_1, k_1, m_2, c_2, k_2\right) \Vert _{\infty } \right] \end{aligned}$$

where $\gamma =\sqrt{k_2/m_2}/\sqrt{k_1/m_1}$ is the tuning ratio, $\Vert G(\omega ) \Vert _{\infty }$ represents the *H-infinity* norm of the receptance frequency response function (FRF) of the primary system, which means its maximum amplitude. Den Hartog addressed this problem nearly a century ago when he proposed the equal-peak method based on the existence of two invariant points independent of absorber damping for the case of undamped primary system, which is widely used in many practical applications today. This method established the foundations of the equal-peak design by providing an approximate solution for stiffness and damping of a tuned mass damper. Surprisingly, an exact closed-form solution to this classic problem has only recently been discovered^21^. The equal-peak design can be extended for cases where the primary system is damped by adding hypothesis that the optimal solution satisfies the condition of two equal peaks about the targeted frequency, but requires numerical solution^28^. In this case, the problem is to find the design variables that will produce equal peaks around the targeted frequency, which depend on the tuning ratio between the host structure and the absorber damping. Although the original formulation of the equal-peak design is based on the minimization of the receptance, it can also be applied for mobility ($M(\omega ) = \dot{X}_1(\omega )/F(\omega )$) and accelerance ($H(\omega ) = \ddot{X}_1(\omega )/F(\omega )$), which are simple variations of the receptance case.

For the purpose of illustration, the receptance of the primary system ($m_1 =$ 10 kg, $k_1=$ $10\times 10^{3}$ $\hbox{N } \hbox{m}^{-1}$ and $c_1=$ 500 $\hbox{N s }\hbox{m}^{-1}$) without the absorber and after its introduction considering different tuning ratios are presented in Fig. 3b. The introduction of the absorber splits the single peak of the primary system without the device into two peaks corresponding to the vibration modes of the coupled system by causing an important reduction in the maximum amplitude of the receptance function and consequently to the vibration of the structure. The optimal solution for the tuning ratio of the absorber assuming mass and damping ratio are constant, is represented by the receptance with two equal peaks and it was obtained by the numerical optimization. Slight deviations from this optimal tuning ratio can significantly degrade the absorber performance by increasing one of the peaks depending on the deviation direction.

The two peaks, designated here by *P* and *Q*, represent respectively the first and second peaks observed about the targeted frequency. Figure 3c shows the behavior obtained by the numerical simulation of amplitude at these two peaks varying according to the tuning ratio, which reveals one point of intersection between the two curves, corresponding to the solution obtained by the equal-peak design. The relation between these two peaks provides information about the optimal design solution. If we represent this relation in a two-dimensional space, as shown in Fig. 3d by plotting the amplitude of the first peak *P* by the second peak *Q*, a simple interpretation of the equal-peak design emerges, represented by the diagonal line where the amplitudes of the two peaks are equal. Hence, the optimal design is achieved along the diagonal line. Design points in the *PQ* space located in the region above the diagonal present a tunig ratio lower than the optimal and those located below the diagonal have a higher tuning ratio. This representation will be further explored in the application of the self-design for the proposed demonstration case.

The solution of the optimization problem expressed in Eq. (2) using deterministic methods is very sensitive to uncertainties which can degrade significantly the attenuation vibration performance of the manufactured vibration absorber. A robust design approach has been proposed to circumvent this issue by formulating the absorber design as a worst-case optimization problem. The key idea is to minimize the maximum response amplitude of the primary system among all possible outcomes over the uncertain set. The formulation of the robust design approach for the lumped mass model illustrated here taking into account uncertainties in the stiffness of the primary system is given by:

3 $$\begin{aligned} \gamma ^{\star } = \arg \left[ \min _{\gamma \in \mathbb {R}^{+}}\left( \max _{ k_1 \in \Delta }\left\| G \left( \omega \mid m_1, c_1,k_1, m_2, c_2, k_2 \right) \right\| _{\infty }\right) \right] \end{aligned}$$

Dell’Elce et al.^21^ addressed this problem recently for a tuned mass damper, where the damping ratio of the absorber is also considered as design variable and they demonstrated that it can be solved numerically using the scenario approach, a general-purpose numerical method for robust optimization^29^. However, it is solved by means of a relaxed version of the problem, because the problem is generally unfeasible in the uncertain domain $\Delta$, which can be a dense set and present an infinite number of constraints. Then, the problem is solved in a feasible subset of $\Delta$ of size defined based on the user-defined risk parameter $\epsilon$, for which, the case $\epsilon =1$ represents the solution of the deterministic problem formulated in Eq. (2) and the case $\epsilon =0^+$ represents the solution of the robust equal-peak design. The scenario approach is used here to solve the problem posed in Eq. (3) using classical optimization algorithms with the associated confidence level $\beta$ in the subset $\Delta _s$ which contain *n* instances or scenarios randomly extracted from the original uncertain set; the solution is only guaranteed with probability $1-\beta$ such that a realization of the uncertain quantities belongs to this subset is larger than a desired threshold $1-\epsilon$^21^.

**Figure 4:**
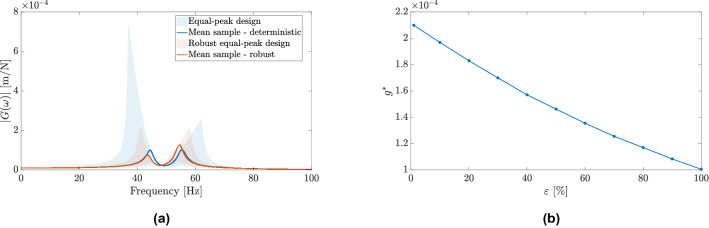
(**a**) Uncertainty propagation effects on the FRF of primary system for the vibration absorber design solution using the equal-peak (deterministic) and robust equal-peak (stochastic) approaches and the mean samples of FRFs. (**b**) Maximum FRF amplitude using the robust optimization scenario approach the as function of the risk level. This function illustrates the trade-off between the performance and robustness in the robust-based approaches.

The robust equal-peak design can significantly reduce a degradation in the vibration attenuation performance due to uncertainties when compared with the deterministic-based equal-peak design, as illustrated in Fig. 4a. In this case, uncertainty was introduced in the stiffness of the primary system by sampling it from a uniform marginal distribution bounded between ± 20$\%$ of its initial stiffness. Although, the robust equal-peak design attenuates the effects of uncertainties in the FRF maximum amplitude, it presents an important drawback illustrated in Fig. 4b, an unavoidable trade-off between the robustness and performance.

## Results

### Self-design manufacturing approach

**Figure 5:**
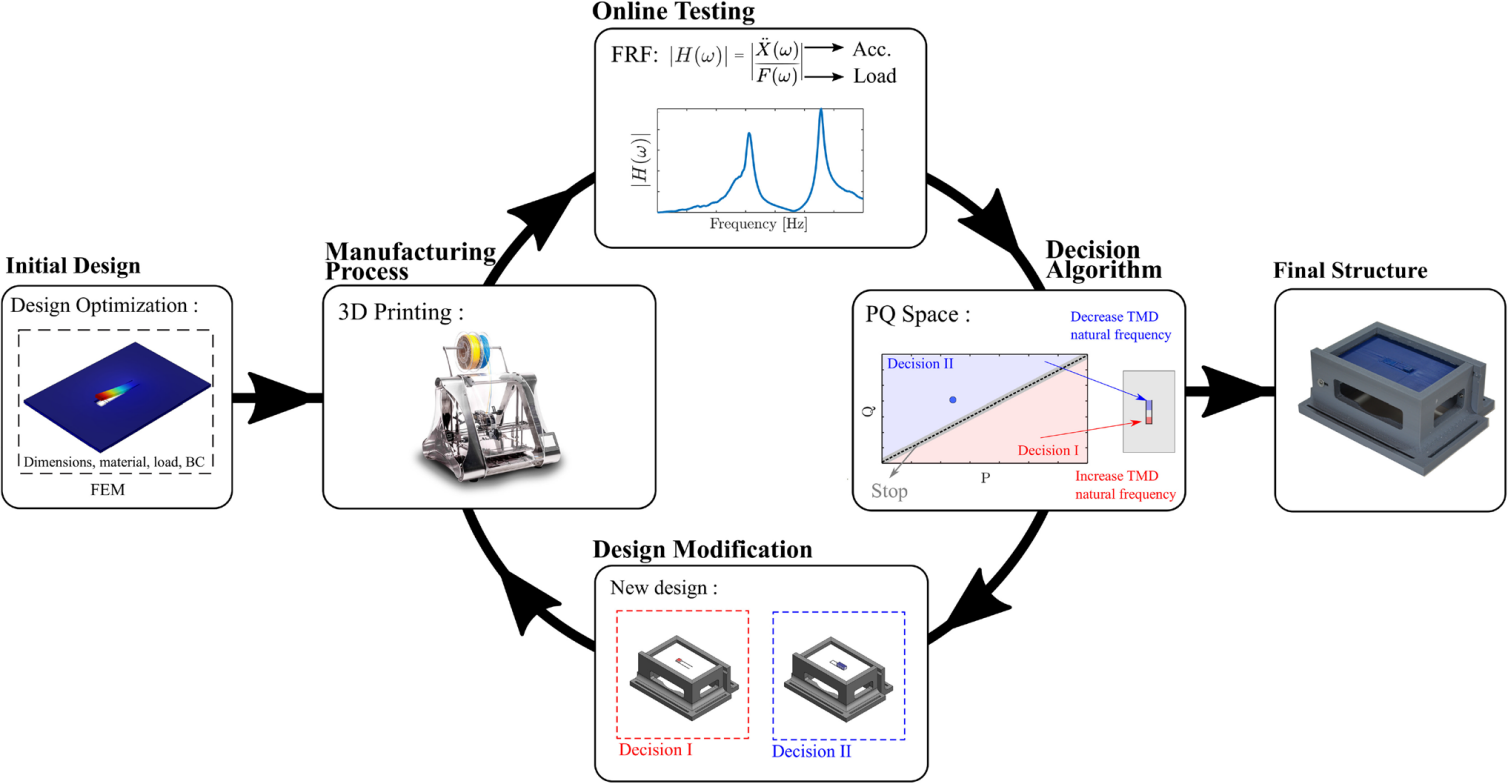
Flowchart of the proposed approach for the application of self-design manufacturing paradigm applied to the design of the vibration absorber.

The flowchart of the proposed approach for application of the self-design manufacturing paradigm in the demonstration case is illustrated in Fig. 5. It starts with the initial design of the structure to be manufactured followed by the main loop containing the four steps of the self-design manufacturing paradigm—manufacturing process, online testing, decision making and design modification—until to obtain the final structure. We discuss in the detail each step of this approach in the following sub-sections.

### Initial design

The proposed approach begins with the initial design of a beam-like absorber on a simply supported beam. The objective function is represented by the accelerance’s maximum amplitude of the plate and the design variable is the length of the beam $\ell$, which can be used to control the working frequency of the vibration absorber. Then, the design optimization problem is defined mathematically as follows:

4 $$\begin{aligned} \ell ^{\star }=\arg \left[ \underset{\mathbf {\ell } \in [L_{min},L_{max}]}{\min } \Vert H(\omega | \ell )\Vert _{\infty } \right] \end{aligned}$$

where $\Vert H(\omega ) \Vert _{\infty }$ represents the H-infinity norm of the accelerance FRF of the plate, and $L_{min}$ and $L_{max}$ are respectively the minimum and maximum length of the beam defined to guarantee the its feasibility.

This problem is addressed by coupling finite element analysis with a gradient-based optimization method to iteratively update the beam length of the absorber from the initial guess to the optimal design, represented by the equal-peak design. The finite element model to simulate the structure was constructed using COMSOL. The plate was modeled with shell-elements with dimensions of $100\times 150\times 3$ mm and the four edges simply-supported. The material properties were assumed to be linear elastic possess Young’s modulus of 2174 MPa, density of 1120 $\hbox {kg}/\hbox {m}^{3}$ and Poisson’s ratio of 0.35, values provided in the data sheet of the ABS material, manufactured by PolyLite. Modal damping values of 1.93% and 2.10% associated respectively with the first and second vibration modes of the structure were assumed based on identification with sacrificial samples.

The sequential quadratic programming algorithm, a gradient-based optimization method, was used to solve the formulated design optimization problem. The objective function was defined as the *H-infinity* norm of accelerance FRF in frequency range selected between 50 and 400 Hz, close to the targeted frequency. The accelerance FRF of the plate considering the measurements at the point indicated in Fig. 5 was obtained by the finite element model simulation. A constraint function was implemented to limit the beam length in the feasible range between 10 and 90 mm. The optimal beam length obtained by the optimization was 46.07 mm, for which the finite element model provides the accelerance FRF with two equal peaks, in agreement with the equal-peak design.

### Integration of manufacturing process and online testing

**Figure 6:**
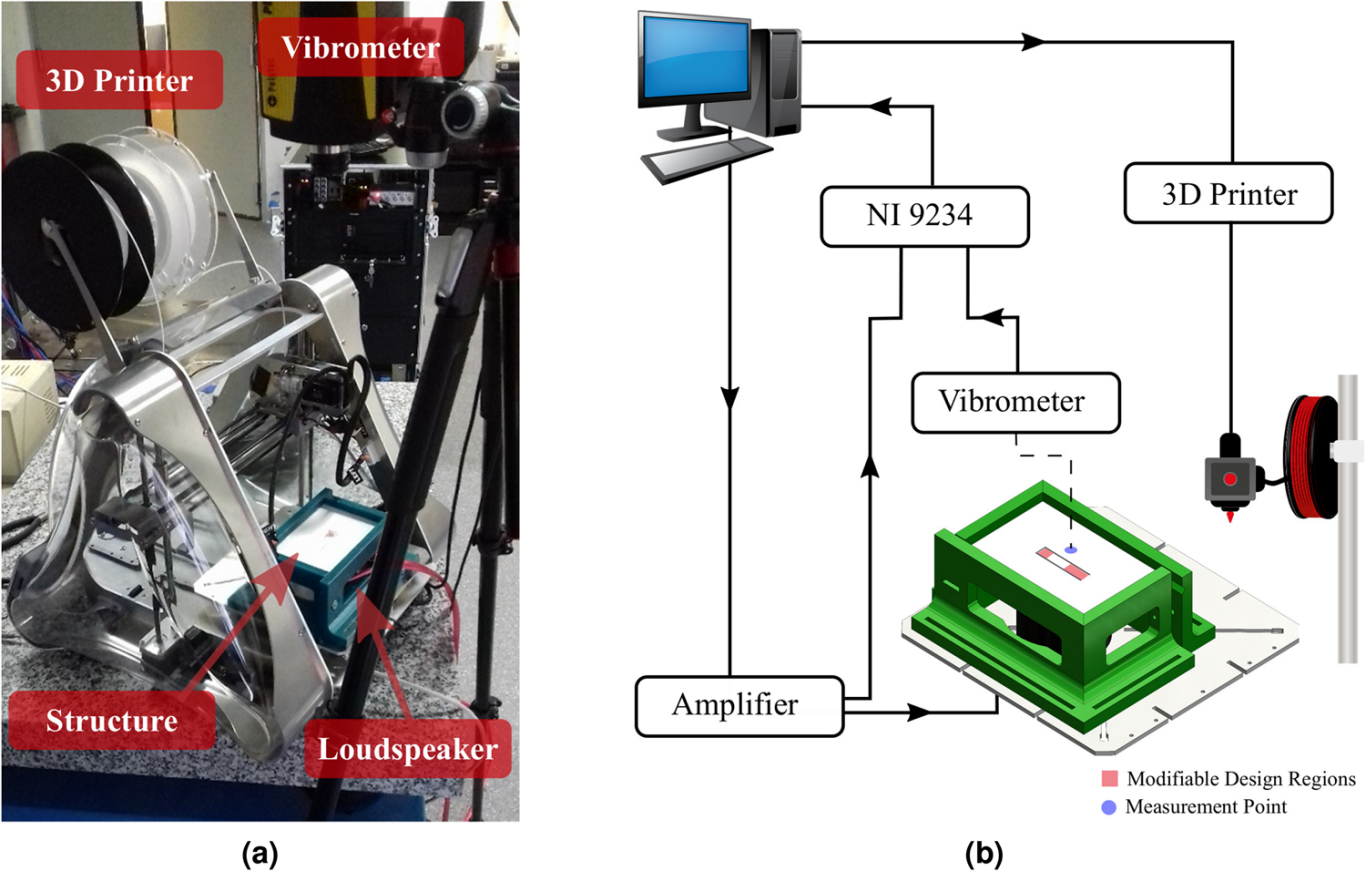
Setup used to implement the self-design approach for the additive manufacturing of the proposed structure. (**a**) Photograph of the system, (**b**) schematic representation of the experimental setup.

The implementation of the proposed approach requires integrating the manufacturing process and the online testing system, so as to allow the real-time performance evaluation of the structure and modifications of the manufactured design. Additive manufacturing by fusion mass modeling with the 3D printer ZMorph model VX was used as manufacturing process. To perform the in situ online testing of the structure inside the 3D printer, a non-contact excitation and acquisition system was coupled to the 3D printer as shown in Fig. 6a. This system consists of a loud-speaker positioned below the structure to excite it and a vibrometer placed on the top of the 3D printer to measure the vibration of the plate at the measurement point indicated.

Figure 6b presents a schematic diagram of the experimental setup. The acquisition and generation of the signals are managed by a MATLAB script. The structure is acoustically excited by the loud-speaker TECTRON using a logarithm sine sweep signal varying from 10 to 1500 Hz generated at a sampling frequency of 10240 Hz by the computer’s sound board and amplified into the BSK-1000 amplifier. The National Instrument board 9234 is used for the synchronized acquisition of the velocity measured by the vibrometer PDV-100 from Polytec and the voltage signal sent to the loud-speaker at the output of the amplifier. The signal processing of input voltage in the loud-speaker and plate’s velocity measured at the measurement point is performed in real-time to estimate the accelerance FRF using a H1 estimator.

The 3D printer communicates with the computer via an ethernet cable using the Telnet protocol. The machine is controlled by the MATLAB script developed to convert the geometry to be printed into the G-code programming language used by the 3D printer. Then, the initial design of the structure can be modified by printing new geometries over the structure. This requires just a calibration step of the initial position of the extruder in a point of reference of the structure. In this demonstration case, the reference point used to calibrate the position of the extruder is the vertex at tip of the beam. The MATLAB script described in this section to manage the experimental setup is available into the github repository https://github.com/jessepaixao/EASER.

### Decision algorithm and design modification

The simple decision algorithm proposed in this demonstration case is based on the equal-peak design represented in the PQ space. Although, the initial design of the structure theoretically yields the maximum performance in vibration attenuation, this optimal point proves inadequate due to uncertainties affecting the manufacturing process, environmental conditions and the model, as well as the absorber high-sensitivity to the mistuning. The decision algorithm will use the experimental evaluation of the structure performance obtained from the online testing system to decide if a design modification could improve the vibration attenuation performance of the absorber in order to achieve the requirements specified by the user.

The equal-peak design provides the condition for the absorber’s optimal performance and it will be the basis for the decision algorithm. In the PQ space, the equal-peak design is represented by the diagonal line in the plane. Then, the main goal of the decision algorithm is to move the point representing the structural design in the PQ space closest to this diagonal line. If the point is close enough based on a user defined threshold value ($T_h$), no design modification is required and the decision algorithm can stop. The diagonal line divides the PQ space into two regions indicated in Fig. 5, one represented by the blue color, associated with the absorber resonance frequency lower than the optimal and another represented by the red color, associated with absorber resonance frequency higher than the optimal. Then, according to the location of the point in the PQ space a decision of the design modification will be defined in order to achieve the main goal.

The problem of improving the vibration attenuation performance of the structure can be seen as the absorber resonance frequency tuning. Because the absorber is a beam, it simplifies the definition of the design modifications to change its resonance frequency. To decrease the resonance frequency of the beam-like absorber we defined the first design modification as adding mass at the free-end of the beam by means of the 3D printing of a $10\times 10$ mm square with a height of 0.2 mm; and in the other sense, to increase the resonance frequency of the beam, we defined the second design modification as increasing the beam rigidity by adding mass in the form of a rectangle of $10\times 20$ mm with a height of 0.2 mm at the beginning of the beam. These two design modifications are illustrated in Fig. 5.

Thus, the decision algorithm can mathematically summarized as follows:

5 $$\begin{aligned} D= \left\{ \begin{array}{ll} \text{ Decision } \text{ I } &{} \text{ if } P > Q + T_h \\ \text{ Decision } \text{ II } &{} \text{ if } P < Q - T_h \\ \text{ Stop } &{} |P-Q| \leqslant T_h \\ \end{array}\right. \end{aligned}$$

### Application of self-design manufacturing approach

**Figure 7:**
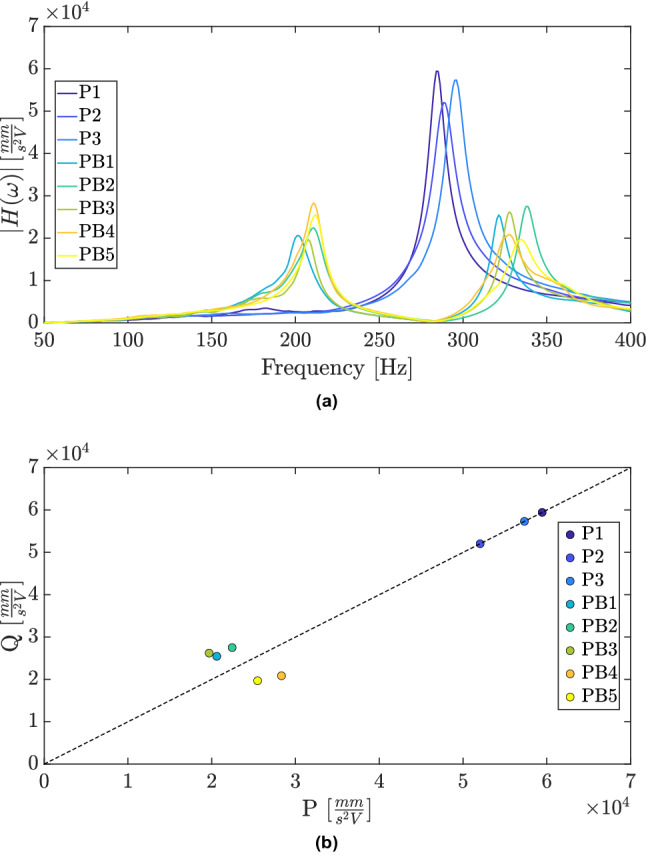
Frequency response function of nominal identical 3D-printed samples of the plate without the absorber and the plate with the beam-like absorber.

The vibration attenuation effect caused by the introduction of the beam-like absorber in the plate structure can be observed in Fig. 7a by comparing the frequency response function of the samples of the plate without the absorber with those with the absorber. The samples of the plate without the absorber are identified as P1, P2 and P3; while the five samples of the plate with the beam-like absorber are identified as PB1, PB2, PB3, PB4 and PB5. Even though the samples manufactured present a unique nominal design, important differences can be observed between their frequency response functions regarding the resonant frequencies and the amplitudes. These differences are due to the uncertainties affecting the manufacturing process, material properties, environmental conditions, boundary conditions, etc. Each manufactured sample can be seen as an unique system that will converge to different equal-peak designs achieved via the self-design approach.

The representation of the data in the PQ space given in Fig. 7b provides a more insightful interpretation of the vibration attenuation performance. As long as the frequency response function of the plate without the absorber has only one peak around the required frequency, it can be represented in PQ space using a one-dimensional space and then in the diagonal line. A comparison between the cluster of points from the samples without and with the absorber reveals an important attenuation of vibration. However, as can be seen, the points representing the plates with absorber are far from the diagonal line, which is a graphical representation of the equal-peak design and then of the optimal performance. This is expected due to uncertainties affecting the process and the high sensitivity of the vibration absorber to mistuning. The major purpose of using the self-design manufacturing approach is to attain the equal-peak design, which is represented visually as pushing the points closer to the diagonal.

**Figure 8:**
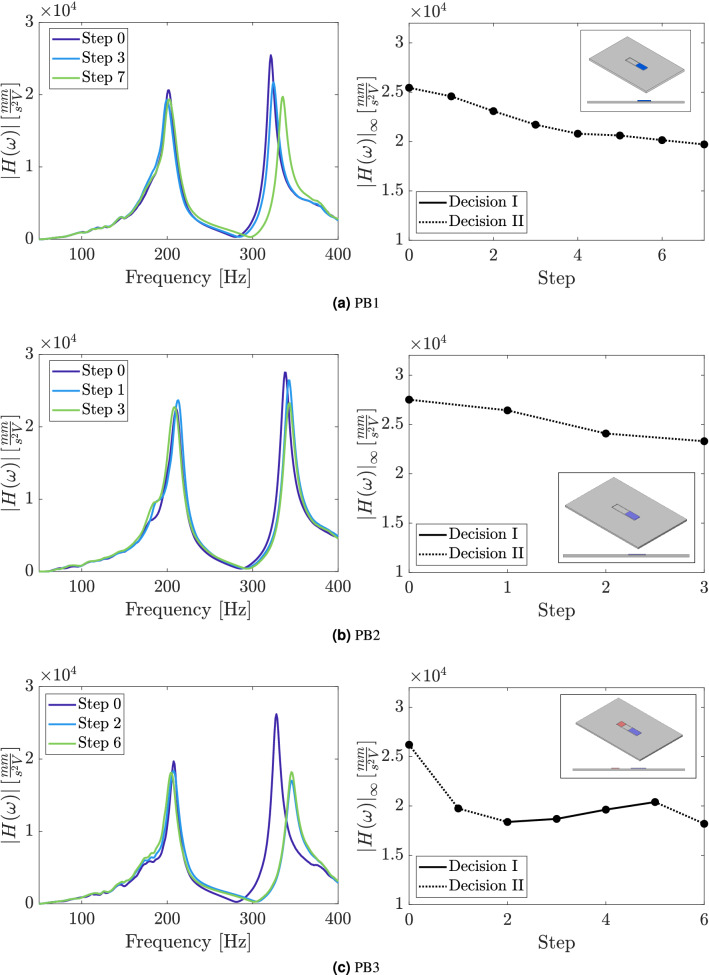

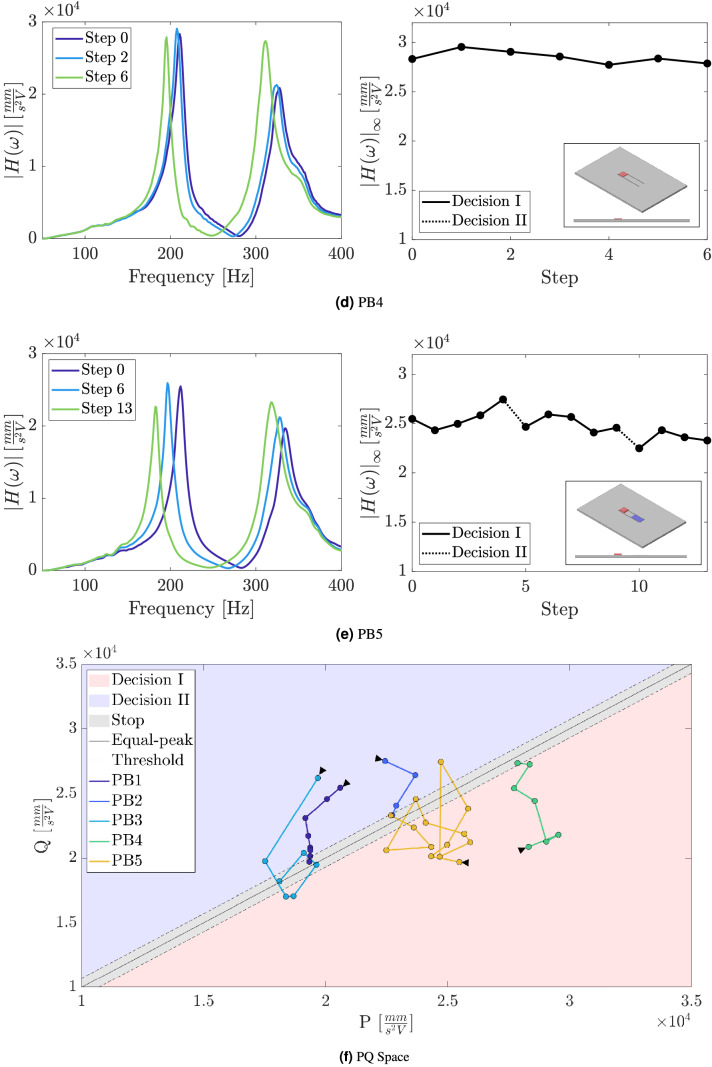
Accelerance FRF and vibration attenuation performance index $|H(\omega )|_\infty$ evolution along the steps of the loop of the self-design manufacturing approach for the samples: (**a**) PB1, (**b**) PB2, (**c**) PB3, (**d**) PB4 and (**e**) PB5. (**f**) Representation of the FRF peaks evolution in the PQ space along the steps of the loop of the self-design manufacturing approach. The decisions made by the decision algorithm in each step are represented by the background colors corresponding to the point location. The threshold value for stop-decision are represented by the dashed diagonal lines and equal-peak design by the solid diagonal line.

The real-time evolution of the application of the self-design manufacturing approach in each one sample with the vibration absorber is presented from Fig. 8a–e by representing the frequency response function of three selected steps and $H_\infty$ along all the steps until the algorithm stop. It can be observed an important reduction in the $H_\infty$ from the initial step to the last step in all samples as the two peaks observed in the frequency response function approach the equal-peak condition. Specifically, the observed percentage reductions in the norm-infinity of the FRFs of each sample are respectively $22.6 \%$, $15.3 \%$, $30.6 \%$, $1.6 \%$ and $8.6 \%$.

The representation of the peaks of the accelerance FRF in the PQ space of samples manufactured presented in Fig. 8f guided the decision algorithm along the main loop of the self-design manufacturing approach. The design modifications introduced in the strucures by using the simple decision algorithm proposed, moved the initial points of the samples into the PQ space to a region closer to the diagonal line, which represents the optimal performance for vibration attenuation of the absorber. Although one could expect a convergence of samples to the same performance and design modifications, it is worth noting that the uncertainties in the manufacturing process affects each sample differently, which explains the differences observed in each sample.

## Discussions

The vibration absorber application was specifically chosen here for its high sensitivity to manufacturing uncertainty and the inherent difficulty of achieving a high performance vibration attenuation with the traditional model-based robust-design approach. In the present case, five nominally identical but different samples served as a starting point for the self-design process. Each starting sample has different dynamic behaviors as seen in Fig. 7a. All structural modifications were restricted to the beam-like absorber and the final designs converged to different beam profiles. Meanwhile, the performance of each sample improved through the iterative closed-loop manufacturing process.

The emergence of the fourth industrial revolution embodying cyber-physical manufacturing technologies provides the necessary ingredients for deploying the proposed self-design manufacturing paradigm. By introducing a developmental plasticity in the manufactured structure, an enhanced performance can be achieved in the presence of uncertainty in the manufacturing process itself. Applications of greater complexity can be envisioned integrating different manufacturing processes, embedded sensors and more sophisticated decision algorithms.

The self-design manufacturing paradigm is particularly attractive for applications whose performance depends on satisfying equality design constraints can benefit from this new paradigm. There is a broad range of engineering applications where the self-design manufacturing paradigm is well adapted, in particular the production of high-performance customized products, including space observation applications, noise and vibration protection devices, sports equipment, musical instruments, medical prostheses, and optimized supports for passive and active metamaterials^30–33^.

## Conclusions

In this paper, we presented a new self-design manufacturing paradigm for engineering applications that closes the loop between design and manufacturing process by leveraging physical intelligence in the form of real-time experimental observations, thus providing design developmental plasticity to the manufactured product. The proposed shift provides a novel strategy to circumvent the unavoidable robustness-performance trade-off of traditional model-based robust-design approaches using a sequential design-manufacturing process. The potential of the proposed approach is illustrated in the design and 3D additive manufacturing of a beam-like absorber structure integrated in a plate whose sensitivity to uncertainty is well-known. The developmental plasticity inherent in the self-design approach yielded tailored designs for each sample, based on a simple decision algorithm, with a concomitant gain in the vibration attenuation ranging from 1.6 to 22.6$\%$. This application demonstrates the effectiveness of the methodology in accounting for manufacturing uncertainties while reducing the dependency on costly high-fidelity physics-based models. The self-design manufacturing paradigm requires a complex integration between the manufacturing process, the online testing system and the decision algorithm for design modification. Future challenges include managing a large number of design degrees of freedom, increasing the number of experimental observation points via full-field measurements or distributed embedded sensors, and decision making under non-unique design paths.

## Data Availability

All the data and code are publicly available at https://github.com/jessepaixao/EASER.
